# The Predictors of Consumer Behavior in Relation to Organic Food in the Context of Food Safety Incidents: Advancing Hyper Attention Theory Within an Stimulus-Organism-Response Model

**DOI:** 10.3389/fpsyg.2019.02512

**Published:** 2019-11-06

**Authors:** Chunnian Liu, Yan Zheng

**Affiliations:** School of Management, Nanchang University, Nanchang, China

**Keywords:** organic products, food safety incidents, cognition, SOR model, the theory of information use environment, hyper attention

## Abstract

Despite the rapid development of China’s organic food industry in recent years, the market size of this industry remains relatively small. Since the organic food market started late in China, consumer groups are mainly concentrated in large cities at present. It is, therefore, urgent to take effective measures to promote the development of China’s organic food market. The current study focuses on the direct and indirect relationships between food safety incidents and organic food purchases by considering the Chinese context, using stimulus-organism-response model and information use environment theory, and introducing a hyper attention cognitive model. The results show that external stimulus (food safety incidents) and internal stimulus (consumer environment orientation) can significantly affect consumers’ response (namely, consumer organic cognition), and the enhancement of consumer organic cognition can promote consumer organic purchase. In addition, consumers’ information environment (the information relating to food safety incidents and environment) can significantly affect their organic food purchase. Moreover, food safety incidents can attract consumers’ hyper attention and thus has a positive impact on consumers’ cognition of organic food. These findings have important implications for research and practice.

## Introduction

In recent years, frequent food safety incidents and environmental problems in China have undermined people’s trust in the traditional food system. In addition, the issues of agricultural pollution are becoming increasingly prominent, due to the overdevelopment and utilization of agricultural resources, the overuse of pesticides and fertilizers in agricultural production, and so on (Xu and Xue, 2015; Ye and Hui, 2016). These problems have prompted consumers to focus on high-quality, safe and more environmentally friendly food (Liu et al., 2013; Hsu and Chen, 2014). Compared with traditional products, organic products are generally considered to have higher nutritional value and are produced in a more natural way without chemicals or harmful pesticides (Wandel and Bugge, 1997; Squires et al., 2001; Pino et al., 2012).

Chinese food is classified differently from foreign food, which is divided into organic food and traditional or conventional food (Yin, 2010). Chinese food, on the other hand, is divided into organic food, green food, and pollution-free food according to its quality grade; of these, organic food has the highest quality grade. The differences between them mainly relate to the production technology. The organic food production process absolutely prohibits the use of chemical synthesis and genetic modification. Unlike with organic food, the other two types of food production allow limited use of synthetic inputs and do not explicitly prohibit the use of genetic engineering technology. Therefore, in Chinese food production, organic food is higher quality, safer and more environmentally friendly than food produced by other processes.

People’s concern for health, environmental protection and safety has resulted in the rapid development of the organic market (Kareklas et al., 2014). The global organic market has reached US$ 81.6 billion in 2015 (Willer and Lernoud, 2017). The organic industry is forecasted to achieve 16% annual growth rate by 2020 (TechSci Research, 2015). In China, the total sales of organic food were about 20–30 billion yuan in 2013, up 1–1.5 times compared with 2007 (Xu, 2017). However, the Chinese organic food market began only in the 1990s, which is comparatively late. As a result, the current market size in China is relatively small, and the total consumption is less than 1 percent of the entire food consumption market; in addition, consumer groups are mainly concentrated in large cities (Xu, 2017). This has undoubtedly hindered the development of organic food in China. Therefore, it is urgent to take effective measures to promote the development of China’s organic food market.

With the increase of public awareness and consumption of organic food, the organic food industry continues to grow worldwide. To maintain this growth, it is necessary to fully understand consumer behavior (Bravo et al., 2013). Researchers have, therefore, begun to pay attention to organic food consumption. Many studies have been conducted on the factors that influence organic food consumption. People with a high demand for organic foods are more convinced that these foods are healthier, tastier, fresher and more environmentally friendly than conventional foods (Wier and Calverley, 2002; Kriwy and Mecking, 2012; Aschemann-Witzel and Grunert, 2015). Buying decisions on organic food are driven largely by personal characteristics, such as freshness, taste, animal welfare, and health benefits (Wier et al., 2008; Yin et al., 2010; Australian Organic Ltd., 2016; Ellison et al., 2016; Rana and Paul, 2017). Consumer attitudes toward food and nutrition are an important factor influencing organic food consumption behavior (Magistris and Gracia, 2008; Aertsens et al., 2009). In addition to food quality, health, taste and other factors affect organic food consumption behavior. Price is also a factor that cannot be ignored, since price is often considered a barrier to organic consumption (Marian et al., 2014), and the premium that is paid for organic food is one of the most important reasons why consumers do not buy it (Magistris and Gracia, 2008; Aertsens et al., 2009). Michaelidou and Hassan (2010) pointed out that consumers’ motivations to buy organic agricultural products include sociocultural reasons (e.g., social image), economic reasons (e.g., price), product reasons (e.g., quality) and personal reasons (e.g., health and safety). In particular, health and environmental concerns, which include the support of sustainable local economic development, have been identified as driving the purchase of organic food (Hamm and Gronefeld, 2004; Yiridoe et al., 2005; Hughner et al., 2007; Schleenbecker and Hamm, 2013).

In addition, the cognition of organic consumption has also been explored by many scholars. Previous studies have shown that the cognition of organic products can promote the purchase of organic food (Kollmuss and Agyeman, 2002; Pagiaslis and Krystallis-Krontalis, 2014). Consumers’ organic cognition can also improve the consistency between their attitudes toward organic products and their buying behaviors (Hidalgo-Baz et al., 2017). Aertsens et al. (2009) showed that providing more information or raising the cognition of organic products can help reduce consumers’ uncertainty about the unique attributes of organic food and alleviate their lack of confidence in certification methods. Such reduced uncertainty may increase the likelihood of a purchase (Thøgersen, 2009). Consumers’ higher organic cognition can also reduce the uncertainty of purchase intention and thus improve the possibility of purchase behavior (Teng and Lu, 2016).

However, the effects that food safety incidents – events of high public concern – have on consumers’ willingness to buy organic food has not yet been well studied. This paper considers the direct and indirect relationships between food safety incidents and organic food purchase by using stimulus-organism-response (SOR) model (Mehrabian and Russell, 1974) and information use environment (IUE) theory (Taylor, 1991). The innovation of this paper is that the hyper attention cognitive model (Hayles, 2008) is introduced, and the relationship between the hyper attention cognitive model and organic purchase is discussed in relation to the Chinese context.

In the next section, we review the previous literature and develop our hypotheses. After the detailed introduction of our research methods and analysis, we present the research results. Finally, we discuss some conclusions, significance and limitations of this study, as well as ideas for further research.

## Theoretical Framework and Hypotheses

### Internal and External Stimulus and Consumer Cognition

According to the SOR theoretical model of environmental psychology, all aspects of the environment play a stimulating role (S), affecting people’s internal states (O), which drives their behavioral responses (R) (Mehrabian and Russell, 1974). The model shows that external environmental factors and conditions influence the mood of the organism, thus prompting the organism to make a behavioral response. It explains that the stimulus of external elements reinforces the internal state of the person (Eroglu et al., 2001). “Organism” refers to the internal states of perception, sensation and thinking (Bagozzi, 1986). Prior studies regarded these constructs as positive and negative effects (Verhagen and Van Dolen, 2011). Finally, people make the final decision and choose their behavioral responses accordingly (Mehrabian and Russell, 1974).

The SOR model is appropriate for the current study because of two reasons. First, the SOR model has been extensively used in previous studies consumers’ behaviors (Parboteeah et al., 2009; Wang et al., 2011; Chang et al., 2014; Luqman et al., 2014; Li and Yuan, 2018). For example, Wang et al. (2011) explored the online store features as stimuli, their effects on consumers’ internal states, and their subsequent impact on consumers’ behavioral responses. Chang et al. (2014) investigated the relationship between consumers’ emotional model and purchase behavior based on the Stimulus-Organism-Response (S-O-R) framework. Second, given the critical roles of environmental cues in influencing consumers’ behaviors, the SOR model provides a parsimonious and structured manner by which to examine the effects of environmental stimuli on consumers’ cognitive or emotional responses and, in turn, their intention to organic purchasing behavior. Therefore, the current study applied the model to consumer behavior.

Based on SOR model, the formation of consumer behavior goes through three stages: internal and external stimulation, psychological activity, and consumer response. First, in terms of internal incentives, consumers’ attitudes toward organic food include environmental orientation and health orientation. Consumers’ environmental motivation and health motivation are positively correlated with their organic cognition (Kareklas et al., 2014). Compared with traditional products, organic products are generally considered to have higher nutritional value and to have been produced in a more natural way without chemicals or harmful pesticides (Wandel and Bugge, 1997; Squires et al., 2001; Pino et al., 2012). Organic products are also considered to be more environmentally friendly (Hughner et al., 2007). In terms of external stimuli, consumers are affected by food safety incidents. In recent years, repeated food safety incidents in China have made consumers more concerned about food safety (Hsu and Chen, 2014). Those who worry about food safety will seek safer food to avoid ingesting harmful substances. According to Wilkins and Hillers (1994), one of the reasons why consumers buy organic food is their perception that non-organic food sold in the market may contain chemical substances or pesticide residues that they wish to avoid. Bezençon and Blili (2010) emphasized in their study that the risks associated with products can influence the degree of consumer participation. Therefore, the effect of internal and external stimuli on consumer cognition has an important impact on consumers’ response behaviors, such as purchase, feedback, and comments.

Previous literature has analyzed the cognition of organic products as a factor in explaining organic or green buying behavior (Kollmuss and Agyeman, 2002; Mostafa, 2007; Pagiaslis and Krystallis-Krontalis, 2014). It has been found that consumers’ experience of food safety incidents may promote organic food purchases. Consumers may produce psychological changes (cognition) and form behavioral responses (purchase behavior) as a result of the effect of internal stimuli (environment orientation and health orientation) and external stimuli (food safety incidents). Therefore, Based on SOR model, the formation of organic purchasing behavior of consumers may go through three stages: internal and external stimulation (environment orientation, health orientation and food safety incidents), psychological activity (cognition), and consumer response (purchase behavior). We propose the following hypotheses:

H1a: Food safety incidents (FSI) have an impact on consumer organic cognition.
H1b: Consumer environment orientation (EO) has an impact on consumer organic cognition.
H1c: Consumer health orientation (HO) has an impact on consumer organic cognition.
H1d: Consumer organic cognition has an influence on consumer organic purchase.

### Food Safety Incidents, Consumer Organic Cognition, and Consumer Organic Purchase

Taylor proposed IUE theory in 1991. The theory presents an analytical framework, centering on users and situations, and including four elements: users and their characteristics, environment, problems to be solved, and solutions to problems. These elements can influence the inflow and outflow of information for any entity and determine the criteria for information value judgment. IUE theory’s identification of factors influencing the use of information provides an important basis for research on situation-based information behavior. Durrance et al. (2006) demonstrated that IUE can be used as a theoretical framework for the study of daily information behavior. IUE theory has been empirically tested in research on the information behavior of social roles, such as medical staff, managers, consumers, and community workers, and studies have proved that it can provide a complete analytical framework for research on situational information behavior (Olatokun and Ajagbe, 2010). Many information behavior researchers have absorbed the concept of IUE in their work (Choo, 1995). The essence of Taylor’s analytical framework is its framing of conditions associated with information use, and Taylor’s analytical framework emphasizes that the outcome of information use is a change in the individual’s capacity to act (Olatokun and Ajagbe, 2010; Shim and Park, 2015). For consumers, they adopt different behaviors according to their characteristics and information environment. Therefore, as far as consumer organic purchasing behavior is concerned, it is exactly in line with the basic elements of the information use environment theory framework. And thus this study applied IUE theory to consumer behavior.

In relation to consumer organic purchasing behavior, IUE theory suggests that the characteristics of consumer environment protection orientation and health orientation result in consumers adopting a solution (i.e., organic purchases) to the problem of frequent food safety incidents. Hill and Lynchehaun (2002) argued that consumers with health and environment orientations believe that organic food can improve their health, because they tend to regard organic food as being more nutritious than non-organic food. Consumption of organic food is also thought by organic consumers to form a healthier diet. Organic food is oriented toward health and environmental protection (Zanoli and Naspetti, 2002; Baker et al., 2004; Essoussi and Zahaf, 2008; Bauer et al., 2013), with the result that the organic market has experienced huge growth (Kareklas et al., 2014). Repeated food safety incidents in China have increased consumers’ consumption of safer, more environmentally friendly and healthier food (Liu et al., 2013; Hsu and Chen, 2014). Therefore, we propose the following hypotheses:

H2a: Food safety incidents (FSI) have a positive impact on consumer organic purchase.
H2b: Consumer environment orientation (EO) has a positive impact on consumer organic purchase.
H2c: Consumer health orientation (HO) has a positive impact on consumer organic purchase.

### Consumer Hyper Attention and Consumer Organic Cognition

Katherine Hayles, an American scholar, proposed the cognitive model of “hyper attention” in 2008. The model is characterized by the constantly shifting focus among multiple tasks, the preference for multiple information flows, the pursuit of strong stimulation levels, and the extremely low tolerance for monotonous boredom. In contrast, the traditional cognitive model of “deep attention” is characterized by a focus on a single target for a long time, ignoring of external stimuli, preference for a single information flow, and high endurance in maintaining the duration of focus (Hayles, 2015). The hyper attention cognitive model is more closely related to the context of the era of new media. With the large-scale popularization and application of the Internet, the types of media have significantly developed and expanded, and the diversification of information transmission modes allows the audience to get rid of the shackles of previous channels.

In recent years, the cognitive model of “hyper attention” has been used in culture research and education research (Zhou, 2014; Zhang, 2017). For example, Zhang (2017) explored the influence of students’ hyper attention cognitive model on traditional students’ teaching mode under the background of “new media”, and how to improve traditional students’ teaching mode. With the rapid development of modern information technology in China, consumers (especially those in economically developed areas) can skillfully take advantage of the convenience of the rich media era to quickly obtain their own desired information, and they can respond flexibly to contemporary society. And thus the cognitive model of consumers may be turning traditional cognitive model of “deep attention” into the cognitive model of “hyper attention” in “New Media” context. Therefore, the current study introduced the hyper attention cognitive model into consumer behavior.

Based on the hyper attention cognitive model, the occurrence of food safety incidents can generate a strong stimulus in consumers, prompting them to pay more attention to food safety and promoting organic cognition. Getting more information about the organic food market may lead to more cognition of organic food and thus promote the purchase of organic food (Magistris and Gracia, 2008). The importance of food safety and the environment is considered the main reason why Chinese consumers buy organic food (Sirieix et al., 2011). In addition, several studies have reviewed studies on organic marketing and concluded that consumers often associate organic food with health, safety and environmental protection (e.g., Yiridoe et al., 2005; Hemmerling et al., 2015).

Based on the hyper attention cognitive model, this paper therefore proposes the following hypothesis:

H3a: Consumer characteristics (CC) have a positive impact on consumer organic cognition.
H3b: Consumer characteristics (CC) have a positive impact on consumer organic purchase.

**FIGURE 1:**
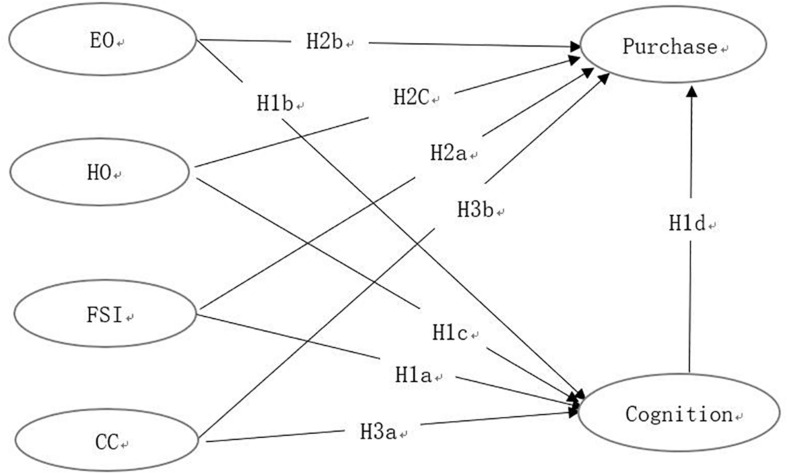
Research framework.

Thus, using SOR model, IUE theory and hyper attention as the research framework, the current study uses partial least square (PLS) to build a research model of consumers’ organic purchasing behavior (see Figure 1).

## Data Sources, Definition of Variables, Research Method

### Data Sources

In order to verify the proposed research hypotheses, the current study conducted an online survey among people with regular jobs in Beijing, Shanghai, Guangzhou and Shenzhen (these were the top-four ranked cities for per capita disposable income in China, according to the China Statistical Yearbook 2018)^1^. Pre-inspection prior to major data collection ensured the comprehensibility of survey items and the appropriateness of data collection procedures. We commissioned the professional online questionnaire service company, Wenjuanxing^2^. An ethics approval was not required as per applicable institutional and national guidelines and regulations of China and the informed consent of the participants was implied through survey completion.

After excluding outliers in the study, we used data collected from 321 consumers out of 369 initial responses. The demographic profile of the sample is shown in Table 1. The number of people aged between 20 and 30 accounted for 51.4% of the total sample, and the number of people aged between 20 and 40 accounted for 88.5% of the sample. Respondents with junior college or undergraduate education accounted for 79.4% of the sample. Respondents with per capita monthly income of Ɏ8,000 to Ɏ12,000 accounted for 34.9% of the sample, and the number of people with per capita monthly income of more than Ɏ12,000 accounts for 33.3% of the sample.

**TABLE 1:** Demographic profile of the sample (*N* = 321).

	***n***	**%**		***n***	**%**
1. Gender			4. Per capita monthly income		
Male	159	0.495	Less than Ɏ3000	2	0.006
Female	162	0.505	Ɏ3000-Ɏ5000	28	0.087
2. Age			Ɏ5000-Ɏ8000	72	0.224
20–30	165	0.514	Ɏ8000-Ɏ12000	112	0.349
30–40	119	0.371	More thanɎ12000	107	0.333
40–50	33	0.103	5. Distribution area of respondents		
50–60	3	0.009	Beijing	94	0.293
Above 60	1	0.003	Shanghai	116	0.361
3. Education			Guangdong Province (including only Guangzhou and Shenzhen)	111	0.346
Below junior high school	0	0			
Junior high school	0	0			
High school or technical secondary school	14	0.044			
Junior college or undergraduate	255	0.794			
Postgraduate and above	52	0.162			

### Definition of Variables

The information sought in this study was consumers’ environment orientation, consumers’ health orientation, food safety incidents, organic cognition of consumers, and organic purchasing behaviors. A five-point Likert scale (1 = “very unconcerned” and 5 = “very concerned”) applied to all the items. Consumer characteristics, including age, income and education, were measured by ranking them on a scale of 1 (“low”) to 5 (“high”). The following scales were used:

(1) Food Safety Incidents (FSI): This scale was mainly derived from previous research by Roddy et al. (1996) and Soler et al. (2002). Three items were used to assess respondents’ concern about food safety incidents. Higher values indicate a stronger concern for food safety incidents. Example items include: “I’m very concerned about food safety incidents,” and “I am well aware of the recent food safety incidents in China.”
(2) Environmental Orientation (EO) and Health Orientation (HO): We used six items adapted from the “self-perception” scale of Cornelissen et al. (2008) and “food values” scale of Lusk (2011) to measure consumer orientations. Hence, we consider orientations as consumers’ self-perception about their past behavior relating to the different benefits of organic products. Specifically, we extracted two orientations: environmental orientation and health orientation. Higher values indicate a stronger concern for the environment or health. Example items include: “When I buy food, I am concerned about the extent to which food affects the environment,” and “I’m very concerned about my own health.”
(3) Cognition: The four-item scale of Cheung and To (2011) was modified and used to assess respondents’ levels of cognition on organic food. Example items include: “I’m very knowledgeable about organic food standards,” and “I’m very knowledgeable about the organic food production process.”
(4) Purchase: This study used the frequency of actual organic food purchases directly to access purchase behavior.

### Research Method

In order to measure the reliability and validity of the variables proposed in this study and to verify the model, we used structural equation modeling software for analysis. At present, structural equation modeling software was divided into two types on the basis of calculation, namely PLS-SEM and CB-SEM. Most researchers considered the two approaches to SEM as complementary, a fact that has already been stressed by the two originators of PLS-SEM and CB-SEM, Jöreskog and Wold (1982).

In recent years, PLS-SEM has been used widely in information management, and the use of PLS-SEM is increasing popular in other fields (e.g., market research and consumer behavior research) (Bontis et al., 2007; Hair et al., 2012). Moreover, PLS is the only model for processing simultaneous both reflective and formative indicators (Chin, 1998; Chin and Newsted, 1999). Many studies showed that PLS-SEM results were robust if data were highly skewed, also when formative measures were used and a one-item measure was used (Ringle et al., 2009). Therefore, this current study used SmartPLS 2.0 to examine the proposed model. In addition, PLS uses different resampling programs to test whether the estimation and path coefficient are significant; in this study, we used “bootstrapping” methods of analysis (Chin, 1998).

The five constructs of FSI, EO, HO, Cognition, and Purchase were measured by the concept of reflective indicators, while Consumer Characteristics (CC) was measured by a formative indicator. The formative construct of CC was mainly derived from previous research by Heise (1972) and MacKenzie et al. (2005). CC is caused by three measures: education, per capita monthly income and age.

### Common Method Variance

As with all self-reported data, there is a potential for common method variance resulting from multiple sources such as consistency motif and social desirability (Podsakoff et al., 2003; Richardson et al., 2009). Several techniques can be used for detecting and controlling the common method variance, such as measured marker variable (correlation-based, regression-based, and CFA-based) and unmeasured latent method factor (Ylitalo, 2009; Podsakoff et al., 2012). We took a statistical approach suggested by Ylitalo (2009) to address concerns regarding common method variance. Ylitalo J. recommended that a factor representing the source of variance due to common method was added to the population model. Therefore, in the present study concerned using marker variables in controlling common method variance, and thus a set of marker variables was created. The indicators were designed to reflect the common method variance in the model, and they were set weakly correlated with all the other indicators in the model except for the correlation caused by the method factor.

**FIGURE 2:**
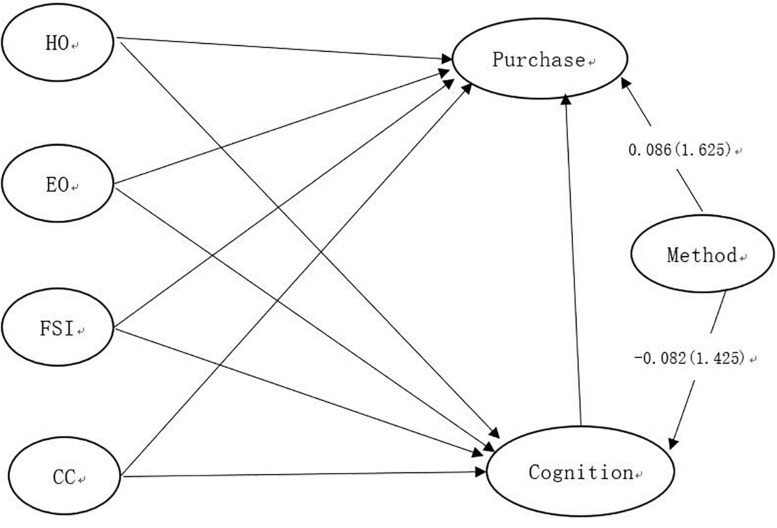
The PLS model for assessing common method variance.

Figure 2 showed that path coefficients of the method factor had insignificant effects on Cognition and Purchase. The method factor included three items from the current survey. Items include: “Degree of addicting to certain food,” “Degree of feeling happy when eating certain food,” and “Degree of considering flavor when buying food.” According to Ylitalo (2009) evidence of common method variance can be obtained by examining the statistical significance of path coefficients of the method factor. If the path coefficients of the method factor are insignificant, we can conclude that common method bias is unlikely to be a serious concern for this study.

## Results

### Measurement Model

In the analysis of the measurement model, PLS can generate weights and loadings at the same time. In general, the weight value is more suitable to interpret formative indicators, while the loading value is more suitable to interpret reflective indicators (Chin, 1998). Because this study dealt with both reflective and formative constructs at the same time, both weights and loadings were generated.

Table 2 shows the loadings and *T*-values of the reflective indicators. Each item has statistical significance. Table 3 shows the weights and *T*-values of the formative indicators, with the significance test carried out by a *t*-test, from which the suitability of this indicator for the potential variable can be examined. The larger the *T*-value is, the stronger it is (Diamantopoulos et al., 2008). The *T*-value in Table 3 indicates the explanatory power of this indicator at different levels of significance. Among them, age and per capita monthly income have statistical significance, while the degree of education has no statistical significance in explaining the potential variable of CC.

**TABLE 2:** Loadings and *T*-values of reflective indicators.

**Variables**	**Items**	**Loadings**	***T*-values**
FSI	1. Degree of concern about food safety incidents	0.891	40.483^∗∗∗^
	2. Degree of understanding food safety incidents in China in recent years	0.874	36.911^∗∗∗^
	3. Degree of anxiety after a food safety incident	0.724	14.980^∗∗∗^
EO	1. Degree of concern about the environment	0.953	85.632^∗∗∗^
	2. Degree of environmental friendliness your behaviors are	0.650	9.621^∗∗∗^
	3. Degree of concern when buying food about the extent to which food affects the environment	0.873	30.827^∗∗∗^
HO	1. Degree of thinking that behaviors are responsible for an individual’s health	0.835	28.201^∗∗∗^
	2. Degree of considering the health effects when buying food	0.863	37.878^∗∗∗^
	3. Degree of care about your own health	0.957	129.413^∗∗∗^
Cognition	1. Degree of knowing about organic food standards	0.832	44.666^∗∗∗^
	2. Degree of knowing about organic food production process	0.842	45.977^∗∗∗^
	3. Degree of understanding the difference between organic and other foods	0.837	49.529^∗∗∗^
	4. Degree of knowing about organic food certification	0.835	43.498^∗∗∗^
Purchase	Frequency of organic food purchases	1.000	\

*^∗∗∗^*p* < 0.001.*

**TABLE 3:** Weights and *T*-values of formative indicators.

**Variable**	**Items**	**Weights**	***T*-values**
CC	Age	0.416	2.181^∗^
	Per capita monthly income	0.872	7.533^∗∗∗^
	Education	–0.185	0.874

*^∗^*p* < 0.1; ^∗∗∗^*p* < 0.001.*

To assess the reliability and validity of reflective constructs, the measurement model was evaluated by examining reliability, average variance extracted (AVE), and discriminant validity (Henseler et al., 2009; Chin, 2010).

In a reflective measurement model, the first criterion is to check the internal consistency and reliability (Henseler et al., 2009). Cronbach’s alpha is often used to estimate for reliability. However, composite reliability, also known as Dillon–Goldstein’s rho (Esposito Vinzi et al., 2010), is considered to be a better indicator of the internal consistency and reliability than Cronbach’s alpha (Chin, 1998). The recommended value for both Cronbach’s alpha and composite reliability is 0.7 or higher (Nunnally, 1978). AVE is used for the evaluation of validity. AVE estimates the amount of variance that a construct captures from its manifest variables or indicators relative to the amount due to measurement errors (Barroso et al., 2010, p. 434). The value of AVE is recommended to be larger than 0.5 (Fornell and Larcker, 1981). As can be seen from Table 4, the values of Cronbach’s alpha and the composite reliability are both higher than 0.7, and all values of AVE are larger than 0.5. Hence, we concluded that all the constructs have good internal consistency and reliability, and that the convergent validity was confirmed.

**TABLE 4:** AVE, Cronbach’s alpha and composite reliability.

**Constructs**	**AVE**	**Cronbach’s alpha**	**Composite reliability**
FSI	0.694	0.794	0.871
EO	0.697	0.785	0.871
HO	0.785	0.861	0.916
Cognition	0.700	0.857	0.903
Purchase	1.000	1.000	1.000

Discriminant validity refers to the degree to which the construct is dissimilar to other constructs. To assess the discriminant validity, we applied the Fornell–Larcker criterion and checked the cross-loadings. For the Fornell–Larcker criterion, the square root of the AVE for each construct should be higher than the correlations between the construct and all other constructs (Fornell and Larcker, 1981). Table 5 presents the square root of AVE and the correlation matrix for constructs. The result shows the square root of the AVE for each construct is higher than the correlations between the construct and all other constructs. In addition, we also used another criterion, cross-loadings, to check for discriminant validity at the item level. It was suggested that the outer loading of each indicator should be larger than all of its cross-loadings (Chin, 1998, 2010). Table 6 shows the outer loadings and cross-loadings in our measurement model. It can be seen that the outer loading of each indicator is larger than all of its cross-loadings and that all indicators have outer loadings higher than 0.6.

**TABLE 5:** Fornell–Larcker criterion.

	**Cognition**	**EO**	**FSI**	**HO**	**Purchase**
Cognition	0.836				
EO	0.366	0.835			
FSI	0.438	0.267	0.833		
HO	0.310	0.427	0.349	0.886	
Purchase	0.536	0.373	0.360	0.277	1.000

**TABLE 6:** Table Cross-loadings.

	**Cognition**	**EO**	**FSI**	**HO**	**Purchase**
EO1	0.342	**0.953**	0.240	0.386	0.330
EO2	0.177	**0.650**	0.151	0.359	0.173
EO3	0.355	**0.873**	0.257	0.353	0.383
FSI1	0.288	0.186	**0.891**	0.244	0.270
FSI2	0.506	0.231	**0.874**	0.349	0.360
FSI3	0.198	0.268	**0.724**	0.250	0.233
HO1	0.281	0.404	0.241	**0.835**	0.207
HO2	0.280	0.333	0.354	**0.863**	0.246
HO3	0.266	0.400	0.328	**0.957**	0.281
Cogntion1	**0.832**	0.341	0.410	0.262	0.463
Cognition2	**0.842**	0.311	0.315	0.262	0.420
Cognition3	**0.837**	0.311	0.391	0.312	0.455
Cognition4	**0.835**	0.259	0.341	0.196	0.451
Purchase	0.536	0.373	0.360	0.277	**1.000**

*The outer loading of each indicator is illustrated in bold font.*

To sum up, the required quality of reliability and validity was confirmed in the measurement model. Consequently, the evaluation of the structural model could proceed further.

### Structural Model

**FIGURE 3:**
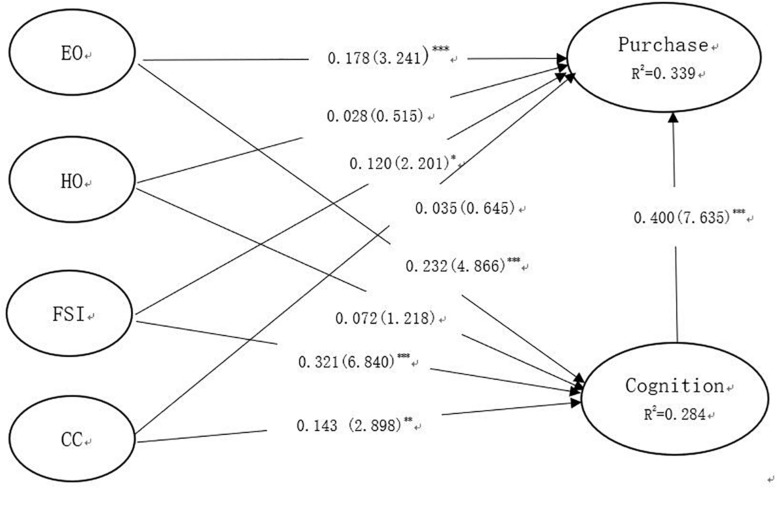
Structural model analysis. ^∗^*p* < 0.1; ^∗∗^*p* < 0.01; ^∗∗∗^*p* < 0.001.

The structural model aims to examine the relationship among a set of dependent and independent constructs. We tested the amount of variance explained and the significance of the relationships. Additionally, a bootstrap resampling approach is recommended to estimate the precision of the PLS estimates (Barroso et al., 2010; Chin, 2010). A bootstrap analysis with 1,000 bootstrap samples (Chin, 1998) was performed to examine the significance of the path coefficients. The result of our structural model analysis is presented in Figure 3.

*R*^2^ refers to the square of correlation between the endogenous variable and the exogenous variable. *R*^2^ values of 0.67, 0.33, and 0.19 for the endogenous constructs in the structural model can be described, respectively, as substantial, moderate, and weak (Chin, 1998; Henseler et al., 2009). As can be seen from Figure 3, the *R*^2^ value of cognition was 0.284, and the *R*^2^ value of purchase was 0.339. The results of this study show that the explainable variation did not reach 67%, but that it also had a certain influence.

The significance of each path coefficient can also be seen in Figure 3. Both FSI and EO had significant effects on Cognition at a significant level of 0.1%, which supports H1a and H1b. Cognition strongly affected Purchase, with path coefficients at 0.400, in support of H1c. FSI and EO were found to have influence on Purchase, with path coefficients at 0.120 and 0.178, respectively, which supports H2a and H2b. CC had significant effects on Cognition, in support of H3a. However, the influence of HO on Cognition and Purchase was not significant, and CC had no significant effects on Purchase.

## Discussion

Referencing the SOR model, using information use environment theory and introducing a hyper attention cognitive model, we studied the direct and indirect relationships between food safety incidents and organic food purchases by considering the Chinese context. Regarding the research process, we developed the research framework and a number of hypotheses by synthesizing the results of previous studies. PLS-SEM verified the constructs, paths, and hypotheses. The major findings of this study are as follows.

Chang et al. (2014) investigate the relationship between consumers’ emotional model and purchase behavior based on SOR model. And they verify that both esthetic formality and esthetic appeal influence purchase behaviors through the emotional model. The results of the current study verify the direct and indirect relationships between food safety incidents and organic food purchase based on SOR model. Firstly, the results of this empirical study confirm that food safety incidents and environmental orientation are positively related to organic cognition, which is consistent with previous research results (e.g., Hughner et al., 2007; Bezençon and Blili, 2010; Hsu and Chen, 2014). In other words, when consumers are stimulated externally (food safety incidents) and internally (environment orientation), consumer organic cognition is promoted. In addition, promoting consumer organic cognition can increase consumer organic purchase, a finding consistent with previous studies that have analyzed whether the cognition of organic products is an important influencing factor on organic or green purchase behavior (e.g., Kollmuss and Agyeman, 2002; Mostafa, 2007; Pagiaslis and Krystallis-Krontalis, 2014). Therefore, our research indicates a high correlation between organic food purchase behavior and organic purchase cognition.

However, consumer health orientation has no significant impact on consumer organic cognition, which is inconsistent with our predictions. But several studies suggest that young consumers have inadequate knowledge regarding organic food (Sanlier, 2009; Green and Knechtges, 2015), and some studies find that elderly people are more health conscious than the young (Kuhn et al., 2007). Therefore, These might explain this result, for most samples are from young consumers in this study.

In addition, food safety incidents and consumer environment orientation have a direct impact on consumers’ purchasing behavior, which is in line with the results of prior literature (Bauer et al., 2013; Liu et al., 2013; Hsu and Chen, 2014). However, consumer health orientation has no significant influence on consumer organic purchase.

Hughner et al. (2007) note that most research identifies health as the main reason for buying organic food, but other authors find no conclusive evidence of an effect of organic food on people’s health, compared with conventional food (e.g., Williams, 2002; Hidalgo-Baz et al., 2017), which might explain this result. In addition, This inconsistency may be related to the classification of Chinese food, which is different from other countries’ division into organic food and traditional or conventional food (Yin, 2010). Chinese food is divided into organic food, green food, and pollution-free food according to its quality grade, with organic food having the highest quality grade. Moreover, due to the late start of China’s organic food market (1990s) and the small market size at present (Xu, 2017), many Chinese consumers cannot distinguish between green food and organic food. Moreover, Many scholars have found that there is a positive correlation between health orientation of Chinese consumers and green consumption (e.g., Liu and Zhang, 2017; Yao, 2019).

With respect to the hyper attention cognitive model, the empirical results of consumer characteristics have a positive impact on the cognition of organic food, which is exactly in line with the cognitive model of hyper attention proposed by Hayles (2008). Zhang (2017) verifies hyper attention cognitive model of students has great effects on the conventional teaching mode. The results of the current study verify hyper attention cognitive model of consumers have a positive impact on the cognition of organic food. Moreover, food safety incidents are strong stimulus events for consumers. Due to the cognitive mode of hyper attention, consumers are strongly stimulated by food safety incidents, which prompt consumers to pay more attention to food safety and promote the cognition of organic food. This result corresponds with findings of previous studies (Magistris and Gracia, 2008). However, consumer characteristics has no significant impact on consumer organic purchase, which is inconsistent with our predictions. But several studies suggest that an attitude–behavior gap or values–action gap arises, such that consumer express food safety incidents concerns, but those concerns do not translate into purchase behaviors (Akehurst et al., 2012; Van Doorn and Verhoef, 2015), which might explain this result.

## Conclusion

This study has explored the direct and indirect relationships between food safety incidents and organic food purchase by considering the Chinese context, using SOR model and IUE theory, and introducing the hyper attention cognitive model. The results show that an external stimulus (food safety incidents) and an internal stimulus (consumer environment orientation) can significantly affect consumers’ response (namely, consumer organic cognition), and that the enhancement of consumer organic cognition can promote consumer organic purchase. In addition, consumers’ information environment (the information about food safety incidents and the environment) can significantly affect their organic food purchase. Finally, this paper has also found that consumer characteristics have a positive impact on consumer organic cognition. Since food safety incidents can attract consumers’ hyper attention, they can promote consumers organic cognition. This is in line with the new cognitive model of that suggests consumers have hyper attention in the information age.

The results are conducive to the sustainable development of the organic food industry and may enrich the research in this field. In particular, the findings have several implications. First, enhancing the publicity of organic food and improving the level of public awareness about organic food are conducive to the purchase of organic food. Organic enterprises can actively publicize and popularize relevant knowledge through the Internet, television, newspapers, the WeChat social media platform and other media channels, in order to promote the public’s understanding of organic food. At the same time, organic food production enterprises can try to invite the public to visit the organic food production base, so as to promote the concept of organic food to the public and to shorten the distance between consumers and organic food. For example, producers could hold events or devise activities that encourage people to learn more about organic food and to choose products for consumption. In addition, powerful production enterprises can launch eco-tourism programs to attract the public to the world of organic food; such programs would enable people to visit production sites and to learn about organic food. At the same time, government authorities or organic food industry associations can hold regular organic food fairs in cities in order to enhance the public’s organic cognition.

In addition, strengthening the display of food quality signs and labels, popularizing food safety knowledge for consumers, promoting the construction of a food quality safety traceability system, and improving the authenticity, richness and legibility of food quality information can help consumers form better perceptions of quality and risk. Meanwhile, the progress and relevant data of food safety incidents should be reported in timely, transparent and objective ways to eliminate consumers’ confusion and anxiety.

## Limitations and Further Research

First, the measure indicators were mainly adapted from existing literature based on the definitions of the self. Future studies could further validate these indicators or develop some new measurements from other perspectives. In addition, since this study examined only two organic orientations, future studies may consider other factors affecting consumers’ organic cognition and purchase, such as hedonic orientation, price sensitivity and consumer preference. Future research may also explore the effects of situational factors on cognition and organic purchases, such as the emotional contagion effects of friends or neighbors, and time pressure. And future research may also use a neuroscientific approach (for example the analysis of the latence time of response) to evaluate the implicit attitude of the people versus organic food, or versus health food.

Finally, this study has considered only the factor of food safety incidents affecting consumer hyper attention, which may underestimate the stimulating effect of other factors. Future studies may consider other factors affecting consumers’ hyper attention, such as the effect of celebrities and other opinion formers. In addition, the discussion of hyper attention in this paper is not perfect, and future research may consider the scope, mechanism and conditions of hyper attention for further study.

## Data Availability Statement

All datasets generated for this study are included in the article/supplementary material.

## Ethics Statement

Ethical review and approval was not required for the study on human participants in accordance with the local legislation and institutional requirements. Written informed consent for participation was not required for this study in accordance with the national legislation and the institutional requirements.

## Author Contributions

CL constructed the theoretical framework of the manuscript. YZ collected the data and made statistical analysis.

## Conflict of Interest

The authors declare that the research was conducted in the absence of any commercial or financial relationships that could be construed as a potential conflict of interest.

## Footnotes

1 http://www.stats.gov.cn/tjsj/ndsj/

2 https://www.wjx.cn
